# Kidney, the Cinderella Story of Transthyretin Amyloidosis

**DOI:** 10.1016/j.ekir.2025.04.050

**Published:** 2025-05-01

**Authors:** Nelson Leung

**Affiliations:** 1 Division of Nephrology and Hypertension, Division of Hematology, Mayo Clinic, Rochester, Minnesota, USA

**Keywords:** amyloidosis, kidney, proteinuria, renal, siRNA, transthyretin

See Clinical Research on Page 1939

Amyloidosis is a group of heterogeneous diseases characterized by tissue deposition of misfolded proteins. Organ involvement is dependent on the native amyloidogenic protein. Currently, > 40 amyloidogenic proteins have been identified.^1^ Some amyloids cause localized disease in a single location of the body; for example kerato-epithelin amyloid in the cornea, lung surfactant protein amyloid in the lung, and Aβ protein precursor in the brain.^2^ Other amyloidoses are systemic, involving 1 or more organs. The most common systemic amyloidosis is systemic immunoglobulin light chain amyloidosis, which can involve nearly every organ except the brain. In contrast, gelsolin amyloidosis is more selective, mainly affecting the kidney, facial nerves, and cornea. Among the visceral organs, the kidney is the most commonly involved organ in amyloidosis.

Transthyretin (ATTR) amyloidosis is a systemic amyloidosis with many unique features. First, there are 2 types of ATTR amyloidosis as follows: (i) a wild type (ATTRwt), which mainly affects older men and (ii) hereditary (ATTRv) form, which has > 120 mutations documented, and more are being discovered.^2^^,^^3^ This is unusual because majority of the amyloidogenic proteins are either mutated or wild type.^2^ In addition, ATTRv has several endemic areas around the world due to a founder effect. The hot spots are in Portugal, Japan, Sweden, and Brazil.^3^ Finally, ATTR has a characteristic pattern of organ involvement. The heart and nervous system, especially the peripheral nerves are the most common.^4^ ATTRwt is overwhelmingly diagnosed in older (median age of 73 years) men (approximately 90%) who present with cardiac symptoms.^4^^,^^5^ Less than 9% of patients with ATTRwt present with polyneuropathy. Although cardiac symptoms remain the dominant feature in ATTRv, approximately 30% will present with polyneuropathy. In addition, patients with ATTRv who presents with cardiac amyloidosis are slightly younger than those with ATTRwt and 28% are women.^5^ In contrast, patients with polyneuropathy can present much younger, as early as their 30s.^5^^,^^6^ Much of the variability in patients with ATTRv can be explained by genetics, which impacts the presentation and age of first symtpoms.^3^ For example, patients with V30M are more likely to present early with polyneuropathy whereas patients with V122I are more likely to present older with cardiomyopathy. In addition, there are racial differences where the mutation V122I is almost exclusively found in Afro-Caribbean. ATTR deposits can also be found in soft tissue such as carpal tunnel ligaments (ATTRwt >> ATTRv), spinal ligamentum flavum (ATTRwt >> ATTRv), and aortic valve, lung and the eye (ATTRv only).^2^^,^^3^

Interestingly, even though ATTR has been recognized for decades, the kidney was only officially recently recognized as a site of involvement. In the amyloid nomenclature updates published by the International Society of Amyloidosis, the kidney was only listed as an ATTR site since 2024.^2^ The lack of recognition can be shown by the fact that only 2 of the 6 phase 3 and 1 phase 1 trials published in the New England Journal of Medicine reported estimated glomerular filtration rate as baseline characteristics, and none reported proteinuria.S1–S7 Even when the trial with inotersen reported adverse events in the kidney, there was little renal laboratory details. This lack of acknowledgement of the kidney in ATTR was not because of lack of information. Studies from endemic areas (Sweden, Portugal, and Japan) have reported patients with elevated creatinine and proteinuria.^7^ One study from Portugal reported 41 nondiabetic dialysis-dependent patients of which 71% had peripheral neuropathy, and 90% had proteinuria that proceeded the kidney failure.^8^ Twelve kidney biopsies were performed among these patients, and they all showed ATTR amyloid deposits with more extensive deposits in the biopsies performed closer to dialysis. There is even a transgenic mouse model where overexpressing wildtype TTR produced amyloid deposits in 80% of the animals in the heart and/or the kidney.S8

The Webster dictionary defines Cinderella as “one suddenly lifted from obscurity to honor or significance.” It is unclear why the kidney was ignored in ATTR. To be completely transparent, until recently, I was one of the guilty ones. This myth may have been promoted by a study of 16,175 amyloid samples that underwent amyloid typing at the Mayo Clinic, and not a single case of ATTR deposits was found in the 1890 kidney samples, even though ATTR made up 28.4% of the total samples.S9 This was not because of false negative results but that kidney biopsies were not being performed in patients with ATTR even by amyloid experts at major amyloidosis centers. Another possible reason may be explained by the timing of dialysis initiation. The average life expectancy after development of peripheral neuropathy before silencing RNA, antisense oligonucleotide, and stabilizers was 11 years whereas the median time to dialysis initiation after the presentation was 10 years.^7^ By the time most patients had developed chronic kidney disease, they were already at the terminal stage of their disease. This matched well with the patients I saw who all had low grade proteinuria (< 1 g/d), severe heart failure, and limited life expectancy.

Characteristics of the patients with kidney disease depends on the study. Women were more likely to develop kidney disease in a study from Portugal; however, this was not confirmed by a study in France.^7^^,^S10 In Portugal, V30M was a risk factor for kidney involvement whereas it was V122I in the French study. Proteinuria preceded end-stage renal disease in nearly all cases and microalbuminuria can even precede neuropathy in some patients. Finally, no asymptomatic carrier had overt proteinuria or renal impairment.

It was therefore exciting to see that kidney outcomes was the main end point of a study in patients with ATTR by Dang *et al.*^9^ published in this issue of Kidney International Reports. The authors reported the kidney outcomes of patients with ATTRv treated with 3 therapies, namely liver transplantation, TTR stabilizer, or silencing RNA and found superior renal survival in the silencing RNA group (100% vs. 44% in the other 2 groups). Only patients treated with silencing RNA had significant proteinuria reduction, and they had a renal response when other therapies had failed. Interestingly, the pattern of women and the V30M mutation emerged again in this French study although a large proportion of patients were excluded, and demographic information on the excluded patients was not reported.

Why is the kidney suddenly gaining all this attention? Better treatment leading to longer survival may have something to do with it. With modern therapies, patients can now actually survive long enough to develop end-stage kidney disease. Another reason may be the kidney’s ability to prognosticate. A recent study of over 2000 patients with ATTR cardiomyopathy found that a declining estimated glomerular filtration rate was a strong predictor of mortality (hazard ratio: 1.71; 95% confidence: 1.43–2.04, *P* < 0.001).S11 What is remarkable is that the declining estimated glomerular filtration rate was independent of N-terminal pro-brain natriuretic peptide, one of the strongest risk factors for cardiac outcomes. Unfortunately, proteinuria measurements were not available, making it impossible to determine if the cause of the declining kidney function was because of cardiorenal syndrome versus ATTR nephropathy. This just highlights the need for more studies that include the kidney in ATTR. Hopefully, these new findings will renew the interest of the kidney in ATTR and further the research in this area.

## Disclosure

The author declared no competing interests.

## Supplementary Material

Supplementary File (PDF) **Supplementary References**.

## Footnotes

Supplementary File (PDF)

**Supplementary References.**
