# Supplementary material for: Kidney, the Cinderella Story of Transthyretin Amyloidosis
Source: Kidney Int Rep. 2025 May 1;10(6):1622–4. doi: 10.1016/j.ekir.2025.04.050 (PMC12232960; doi:10.1016/j.ekir.2025.04.050)
Supplement: Supplementary File (PDF) — Supplementary References. [file mmc1.pdf]

Supplemental references

S1. Fontana M, Berk JL, Gillmore JD, et al. Vutrisiran in Patients with Transthyretin Amyloidosis with Cardiomyopathy. The New England journal of medicine 2025; 392: 33-44.

S2. Gillmore JD, Judge DP, Cappelli F, et al. Efficacy and Safety of Acoramidis in Transthyretin Amyloid Cardiomyopathy. The New England journal of medicine 2024; 390: 132-142.

S3. Maurer MS, Kale P, Fontana M, et al. Patisiran Treatment in Patients with Transthyretin Cardiac Amyloidosis. The New England journal of medicine 2023; 389: 1553-1565.

S4. Maurer MS, Schwartz JH, Gundapaneni B, et al. Tafamidis Treatment for Patients with Transthyretin Amyloid Cardiomyopathy. The New England journal of medicine 2018; 379: 1007-1016.

S5. Benson MD, Waddington-Cruz M, Berk JL, et al. Inotersen Treatment for Patients with Hereditary Transthyretin Amyloidosis. The New England journal of medicine 2018; 379: 22-31.

S6. Fontana M, Solomon SD, Kachadourian J, et al. CRISPR-Cas9 Gene Editing with Nexiguran Ziclumeran for ATTR Cardiomyopathy. The New England journal of medicine 2024; 391: 2231-2241.

S7. Adams D, Gonzalez-Duarte A, O'Riordan WD, et al. Patisiran, an RNAi Therapeutic, for Hereditary Transthyretin Amyloidosis. The New England journal of medicine 2018; 379: 11-21.

S8. Buxbaum J, Tagoe C, Gallo G*, et al.* The pathogenesis of transthyretin tissue deposition: lessons from transgenic mice. *Amyloid* 2003; **10 Suppl 1:** 2-6.

S9. Dasari S, Theis JD, Vrana JA*, et al.* Amyloid Typing by Mass Spectrometry in Clinical Practice: a Comprehensive Review of 16,175 Samples. *Mayo Clin Proc* 2020; **95:** 1852-1864.

S10. Solignac J, Delmont E, Fortanier E*, et al.* Kidney involvement in hereditary transthyretin amyloidosis: a cohort study of 103 patients. *Clin Kidney J* 2022; **15:** 1747-1754.

S11 Ioannou A, Razvi Y, Porcari A, et al. Kidney Outcomes in Transthyretin Amyloid Cardiomyopathy. JAMA Cardiol 2025; 10: 50-58.
